# Systemic Copper Disorders Influence the Olfactory Function in Adult Rats: Roles of Altered Adult Neurogenesis and Neurochemical Imbalance

**DOI:** 10.3390/biom11091315

**Published:** 2021-09-06

**Authors:** Sherleen Xue-Fu Adamson, Wei Zheng, Zeynep Sena Agim, Sarah Du, Sheila Fleming, Jonathan Shannahan, Jason Cannon

**Affiliations:** 1 School of Health Sciences, Purdue University, West Lafayette, IN 47907, USA; sherleen.adamson@gmail.com (S.X.-F.A.); sena.agim@gmail.com (Z.S.A.); sarah.yang.du@emory.edu (S.D.); jshannah@purdue.edu (J.S.); 2 Purdue Institute for Integrative Neurosciences, Purdue University, West Lafayette, IN 47907, USA; 3 Department of Pharmaceutical Sciences, Northeast Ohio Medical University, Rootstown, OH 44272, USA; sfleming1@neomed.edu

**Keywords:** Cu deficiency, Cu overload, olfactory, subventricular zone, rostral migratory stream, neurochemical homeostasis, adult neurogenesis, GABA

## Abstract

Disrupted systemic copper (Cu) homeostasis underlies neurodegenerative diseases with early symptoms including olfactory dysfunction. This study investigated the impact of Cu dyshomeostasis on olfactory function, adult neurogenesis, and neurochemical balance. Models of Cu deficiency (CuD) and Cu overload (CuO) were established by feeding adult rats with Cu-restricted diets plus ip. injection of a Cu chelator (ammonium tetrathiomolybdate) and excess Cu, respectively. CuD reduced Cu levels in the olfactory bulb (OB), subventricular zone (SVZ), rostral migratory stream (RMS), and striatum, while CuO increased Cu levels in these areas. The buried pellet test revealed both CuD and CuO prolonged the latency to uncover food. CuD increased neural proliferation and stem cells in the SVZ and newly differentiated neurons in the OB, whereas CuO caused opposite alterations, suggesting a “switch”-type function of Cu in regulating adult neurogenesis. CuO increased GABA in the OB, while both CuD and CuO reduced DOPAC, HVA, 5-HT and the DA turnover rate in olfactory-associated brain regions. Altered mRNA expression of Cu transport and storage proteins in tested brain areas were observed under both conditions. Together, results support an association between systemic Cu dyshomeostasis and olfactory dysfunction. Specifically, altered adult neurogenesis along the SVZ-RMS-OB pathway and neurochemical imbalance could be the factors that may contribute to olfactory dysfunction.

## 1. Introduction

Copper (Cu) is an essential element for human health serving as an indispensable cofactor for numerous enzymes and proteins that are widely involved in a number of diverse biochemical reactions [1,2,3,4]. In the central nervous system (CNS), Cu-containing enzymes such as cytochrome C oxidase, superoxide dismutase, dopamine-β-monooxygenase, and tyrosinase, play pivotal roles in various biological processes, such as energy metabolism, iron metabolism, anti-oxidative defense, and synthesis of neurotransmitters and neuropeptides, among others [5,6,7,8]. Nevertheless, by actively interacting with oxygen, excessive free Cu ions can initiate a cascade of reactions leading to the generation of highly damaging hydroxyl radicals. Thus, a dyshomeostasis of Cu, in either the form of Cu deficiency (CuD) or Cu overload (CuO), is deemed disruptive to normal brain function.

Imbalanced Cu homeostasis has been well characterized in two inherited neurodegenerative disorders, i.e., Menkes disease and Wilson’s disease. In Menkes disease, Cu deficiency occurs due to ATP7A mutations while in Wilson’s disease, a genetic defect in ATP7B causes excessive Cu accumulation in the liver and brain [9,10]. A large body of evidence has also implicated a disrupted Cu homeostasis in pathogeneses of neurodegenerative disorders, e.g., Parkinson’s disease (PD), Alzheimer’s disease (AD), amyotrophic lateral sclerosis, prion disease, and Huntington’s disease [11,12,13,14,15]. Our own human studies on manganese exposure-associated Parkinsonian disorder reveal that Cu levels are significantly higher in blood, serum, and saliva samples from manganese-exposed smelters and welders compared to control workers [16,17,18]. Moreover, studies by our laboratory and others on manganese-poisoned animal models (non-human primates and rodents) demonstrate a significant increase of Cu in brain regions including caudate putamen, striatum, hippocampus, and choroid plexus [19,20,21,22]. Thus, Cu dyshomeostasis unequivocally underlies neurodegenerative diseases and disorders.

By using synchrotron X-ray fluorescent microscopy, we have reported an extraordinarily high Cu level in the subventricular zone (SVZ) of the rat brain [23]. Data by atomic absorption spectrophotometry further confirm significantly elevated Cu levels in the SVZ and olfactory bulb (OB) compared to other brain regions [19,24]. Importantly, the SVZ is an active proliferative niche in the adult brain. Limited data have suggested a role of Cu in regulating embryonic stem cell differentiation [25,26,27]. Given the higher levels of proliferation, migration, and differentiation occurring in the SVZ, it seems likely that the high Cu environment may be required to maintain normal energy metabolism and/or other biochemical reactions deemed to be essential to adult neurogenesis. However, there is a clear lack of knowledge on the role and mechanisms of Cu in regulating adult neurogenesis in the SVZ and OB.

During adult neurogenesis, new neurons are generated from two proliferative niches in adult brain, i.e., the SVZ in the lateral ventricles and the subgranular zone (SGZ) in the hippocampus. The SVZ is rich in neural stem/progenitor cells (NSPCs) and functions as the origin of adult neurogenesis [28,29]. The SVZ possesses 4 cell types: (i) ependymal cells facing the cerebrospinal fluid (CSF), which is secreted and regulated by the choroid plexus in brain ventricles, (ii) β-tubulin or Doublecortin (DCX)-positive Type A migratory neuroblasts, (iii) glial fibrillary acid protein (GFAP)-positive Type B astrocytic stem cells, and (iv) Nestin-positive Type C transit-amplifying cells. Newly generated NSPCs in the SVZ can migrate alongside the rostral migratory stream (RMS) to reach the OB [29,30]. On this SVZ-RMS-OB pathway, the proliferating cells further differentiate in adjacent brain regions to supply renewed neurons and to compensate for the loss of neurons in order to maintain normal brain function [28,31].

Loss of neurons in selective brain regions is a pathological characteristic of numerous neurodegenerative diseases including PD. Olfactory impairments, such as deficits in odor identification, detection, and discrimination, are found in patients with PD and AD [32,33,34,35,36]. Olfactory deficits can trigger aberrant adult neurogenesis in the SVZ-RMS-OB system. Previous studies in zebrafish have shown that Cu is capable of inducing olfactory dysfunction, abnormal neurogenesis, and dysfunctional locomotor behavior [4]. Clinical evidence also suggests an olfactory dysregulation in patients with Cu-homeostasis dysfunctions in Wilson’s disease [37,38]. Thus, it has become necessary to investigate the role of Cu in regulating adult neurogenesis in the SVZ, which ultimately affects olfactory function.

Maintaining a balanced cellular Cu homeostasis requires close coordinations among a group of Cu regulatory proteins [6,39]. These include Cu transporter-1 (CTR1) and divalent metal transporter-1 (DMT1) which function to regulate Cu uptake by the cell membrane [40]. Further, there are various isoforms of metallothioneins regulating intracellular Cu storage [6]. These Cu regulatory proteins exist in the mammalian brain with a particular abundance in the brain capillary endothelial cells constituting the blood-brain barrier and in the choroid plexus epithelial cells forming the blood-CSF barrier [6,39,41,42,43]. Our recent data has confirmed the presence of these regulatory proteins in the SVZ [19]. Conceivably, disruption of Cu systemic homeostasis would adversely affect the expression of these Cu regulatory proteins in the Cu-rich SVZ, which would in turn affect normal olfactory function.

The current study was designed to investigate whether alteration of systemic Cu levels, resulting from either Cu deficiency (CuD) or Cu overload (CuO) conditions, has any impact on olfactory function in adult rats through the modulation of adult neurogenesis in the Cu-rich SVZ and OB, and to determine the ensuring alteration of neurochemical balance within the OB. We first established and verified animal models of CuD and CuO, followed by examining alterations in olfactory function. Further, we investigated the mechanism by which CuD or CuO interrupted adult neurogenesis and neurochemical balance in brain regions critical to the olfactory function.

## 2. Material and Methods

### 2.1. Materials

Chemical reagents were purchased from the following sources: mouse monoclonal anti-BrdU antibody from Santa Cruz Biotechnology (Dallas, TX, USA), ProLong Gold anti-fade reagent, Alexa Fluor 488 goat anti-rabbit IgG (H+L) antibody (Catalog No. A11008) and Alexa Fluor 555 goat anti-mouse IgG (H+L) antibody (Catalog No. A21424) from Life Technologies (Carlsbad, CA, USA); anti-doublecortin (DCX) (Catalog No. ab18723) and anti-NeuN (Catalog No. ab177487) antibodies, and chicken polyclonal anti-glial fibrillary acidic protein (GFAP) antibodies from Abcam (Cambridge, MA, USA); anti-tyrosine hydroxylase sheep polyclonal antibody (Catalog No. AB1542) from Millipore (Burlington, MA, USA); anti-GAD67 mouse monoclonal antibody (Catalog No. ab26116) from Abcam (Branford, CT, USA); paraformaldehyde (PFA) from ACROS Organics (Morris Plains, NJ, USA); bovine serum albumin (BSA) from AMRESCO (Solon, OH, USA); and normal goat serum and normal donkey serum from Jackson Immuno Research Labs (West Grove, PA, USA). All reagents were of analytical grade, HPLC grade, or the best available pharmaceutical grade.

### 2.2. Animals and Treatment

Male Sprague–Dawley rats were purchased from Taconic Biosciences (Hudson, NY, USA). At the time of use, the rats were 10-weeks old weighing 220–250 g. Upon arrival, rats were housed in a temperature-controlled, 12/12-h light/dark cycle room and allowed to acclimate for one week prior to experimentation. Rats were provided with deionized water and designated pellet (See details in systemic Cu-deficiency/overload treatments) *ad libitum*. The study was conducted in compliance with standard animal use practices and approved by the Animal Care and Use Committee of Purdue University (Protocol#1112000526 approved on 17 May 2019).

**Figure 1:**
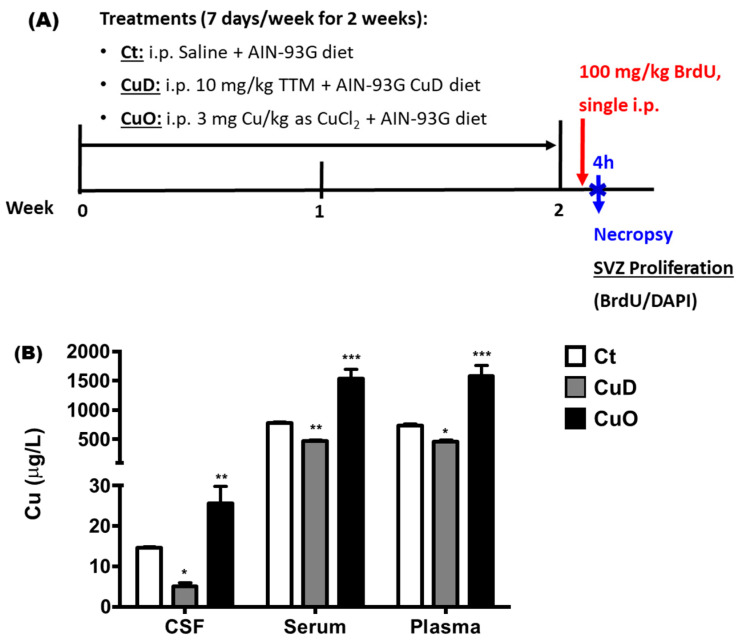
Experimental design and model verification. (**A**). Protocol for 2-week CuD or CuO treatments in rats. Ct: control; CuD: Cu deficiency; CuO: Cu overload. (**B**). Cu concentrations in body fluids. CSF: cerebrospinal fluid. Data represents mean ± SEM, n = 6–7/group. *: *p* < 0.05, **: *p* < 0.01, ***: *p* < 0.001, as compared to the correspondent Ct group, using one-way ANOVA analysis.

The methods to create systemic Cu deficiency (CuD) and Cu overload (CuO) rat models have been extensively published in literature [44,45,46,47,48,49,50,51,52]. In addition to Cu-controlled diet, the CuD and CuO models require ip. injections of a Cu chelator or Cu supplement, respectively. Thus, we modified existing approaches and designed the protocol as illustrated in Figure 1A. Briefly, after a 1-week acclimation period, rats were randomly assigned to control, CuD, or CuO groups. CuD rats had free access to a modified AIN-93G purified CuD diet containing 0.3 ppm Cu (Teklad Diets from Envigo Corp, Indianapolis, IN, USA); CuD rats also received ip. injections of 10 mg/kg of the Cu chelator ammonium tetrathiomolybdate (TTM) once daily for 7 days a week. CuO rats had free access to a normal AIN-93G purified CuO diet containing 6 ppm Cu (Teklad Diets from Envigo Corp, Indianapolis, IN, USA); these rats also received ip. injections of 3 mg Cu/kg as CuCl_2_ once daily for 7 days a week. Control rats had free access to the normal AIN-93G purified diet and received daily ip. injections of saline. All treatments lasted for 2 consecutive weeks. Cu concentrations in serum, plasma, and cerebrospinal fluid (CSF) of treated animals were determined by atomic absorption spectrophotometry (AAS) to verify the effectiveness of CuD and CuO treatments.

To evaluate the impact of systemic Cu dyshomeostasis on cell proliferation in the SVZ described in Section 2.5, at 24 h after the last treatment, rats received a single dose of BrdU (100 mg/kg, ip.) and were necropsied 4 h later (Figure 1).

### 2.3. Buried Pellet Test

Olfactory function was evaluated via the buried pellet test which is based on the time taken to uncover a buried food pellet hidden under the bedding, as described in literature [53,54,55]. In brief, 24 h after the last treatment, rats were placed in a clean cage (48-cm length × 25-cm width × 20-cm height) and provided with several pieces of the pellets (mini marshmallows) for three consecutive days with food restriction to 85–90% of body weight. Rats were then fasted overnight before the test and habituated to the testing room for 1 h with no water bottle and feeder bin but with a filter top lid.

Two clean cages for testing the olfactory function were prepared in the same means with clean bedding at approximately a depth of 5 cm which was evenly distributed through the cage, except that one mini marshmallow was placed 3.8 cm below the bedding in the test cage. The test began by placing the test rat in the clean cage without marshmallow. Following 1 h of habituation, the test rat was removed from the clean cage, placed in the center of the test cage, and the timer was started. The latency to uncover the mini marshmallow was recorded. If the rat did not find the pellet within 5 min, the trial was ended and a score of 300 s was noted. The bedding was emptied from the test cage that was then cleaned with cleaning solution and filled with clean bedding for the next trial.

### 2.4. Brain Tissue Collection

All animals were anesthetized using ketamine/xylazine (75:10 mg/kg, 1 mg/kg ip.). Blood and CSF samples were collected, followed by brain dissection. Brains were removed from the skull, one hemisphere was fixed by 4% paraformaldehyde (PFA) in phosphate-buffered saline (PBS) for 7 days and subjected to immunohistochemistry studies.

The other hemisphere was dissected to harvest brain regions including the olfactory bulb (OB), lateral olfactory tract (LOT) also known as anterior rostral migratory stream (aRMS), subventricular zone (SVZ), choroid plexus (CP), striatum (STR), hippocampus (HP), frontal cortex (FC), and cerebellum (CB). The harvested brain samples were stored at −80 °C freezer for AAS, qPCR and HPLC analyses.

### 2.5. Determination of Cu Concentration by Atomic Absorption Spectroscopy (AAS)

Prior to AAS quantification of Cu levels, brain samples were digested with concentrated ultrapure HNO_3_ in a MARSXpress microwave-accelerated reaction system. Samples of SVZ, choroid plexus, CSF, and plasma were digested overnight with HNO_3_ in the oven at 55 °C. An Agilent Technologies 200 Series SpectrAA with a GTA 120 graphite tube atomizer was used to quantify Cu concentrations. Digested samples were diluted by 50, 500, or 1000 times with 0.1% (*v*/*v*) HNO_3_ in order to maintain a reading within the concentration range of the Cu standard curve. The Copper AAS Standard (1000 mg/L in 5% nitric acid) was purchased from Agilent Technologies (Part #5190-8279). The calibration standard range for Cu was 0–25 μg/L and the detection limit for Cu was 0.9 ng/mL of the assay solution. Intra-day precision of the method for Cu was 1.6% and inter-day precision was 3.7% [19,24,41,56].

### 2.6. Immunohistochemistry (IHC) Staining

Following the fixation with 4% PFA for 7 days, brain hemispheres were dehydrated in 30% sucrose for another 7 days. Serial 30-µm thick coronal or sagittal sections were cut using a microtome and stored in cryoprotectant solution at −20 °C. Specifically, for coronal and sagittal sectioning, the brains were sectioned roughly from Bregma 2.0 mm to −6.0 mm (coronal) and Lateral 0.2 mm to 4.0 mm/hemisphere (sagittal) according to the rat brain atlas in order to cover the entire distance of the SVZ, which yielded about 250–270 sections/brain (coronal) and 125–135 sections/hemisphere (sagittal). All of the coronal sections were placed in a twelve-well plate in serial order, and all of the sagittal sections collected from one hemisphere were placed in 6 wells of the twelve-well plate in serial order. Each well contained about 18–22 sections for both coronal and sagittal sections, which accounted for 1/12 of the total brain sections. The 18–22 sections within the same well and with the same well number order across all animals were then processed for immunohistochemistry (IHC) staining.

Every twelfth section (360 µm interval), covering the distance of the lateral ventricle and OB, was processed for IHC analysis. Sections were incubated with various primary antibodies against GAD67 (1:5000), tyrosine hydroxylase (TH) (1:500), anti-doublecortin (DCX) (1:1000), Nestin (1:500), or NeuN (1:2000) overnight at 4 °C. Free-floating sections were washed with PBS (3 × 10 min/wash), incubated in 2N HCl for 2 h at room temperature, and then blocked in 0.1 M borate buffer for 15 min (pH 8.4). After 3 washes with PBS (10 min/wash), sections were incubated in blocking solution (0.3% Triton X-100, 1% BSA, and 5% normal goat serum in PBS) for 1.5 hr at room temperature, followed by overnight incubation with mouse anti-BrdU (1:500) at 4 °C. The sections were washed with PBS (3 × 10 min) and incubated with Alexa Fluor 555 goat anti-mouse IgG (H+L) antibody (1:500) for 2 h at room temperature. For imaging, sections were further incubated with DAPI (4′,6-diamidino-2-phenylindole) in secondary antibody solution for 15 min at room temperature. All sections were then rinsed with PBS (3 × 10 min) and mounted using Fluorescent Mount G.

### 2.7. Confocal Imaging and Cell Counting

After IHC processing, brain sections were examined using a Nikon TE2000-U inverted microscope equipped with a Nikon A1 confocal system from Nikon Instruments Inc. (Melville, NY, USA). Images were taken using the software NIS Elements AR (v4.20). All images were assembled and labeled in Photoshop CC. For cell counting, all sections were analyzed with appropriate filter or laser combinations under an objective lens of 20×/0.75 (DIC N2, ∞0.17 WD). Large image plus Z-Stack scanning was employed to confine the entire SVZ from coronal.

IHC was performed simultaneously on sections from different groups to detect the target cells. Series of every twelfth section (30 µm thickness, 360 µm apart) through each lateral ventricle were processed. The cell density was determined through a blinded quantitative histological analysis. A profile count method was used; every single BrdU(+) cell including partial of BrdU(+) nuclei at the border of section, or BrdU and DAPI double-labeled cells in the different sub-region of HDG in the multiplanes throughout the entire 30 µm section, were counted under the fluorescent or confocal microscope using the large Z-stacking images through a whole series of sections. Those BrdU/DAPI labeled cells were considered the target cells for cell counting. The total number of quantified cells was justified by correction [57,58]. The total number of BrdU/DAPI labeled cells were then calculated by the following equation:

Total cell number = the sum of actual cell counting number × 12 and expressed as total number/target region/brain (n = 3 or 4 brains for each group).

### 2.8. High-Performance Liquid Chromatography (HPLC)

Neurochemical analysis was performed similarly to described in previous studies [59,60]. Briefly, frozen brain samples were sonicated in 0.5 mL of 0.4 N perchloric acid (HClO_4_) on ice. Samples were then centrifuged at 16,400× *g* for 35 min at 4 °C. The supernatant was transferred to a 0.22 mm Spin-X tube with nylon filter (Corning, Corning, NY, USA) and centrifuged at 1000× *g* for 15 min at 4 °C. Samples were then stored at −80 °C until HPLC analysis.

The HPLC system consisted of a Dionex Ultimate 3000 Model ISO-31000BM pump, a model WPS-3000TBSL autosampler, Coulochem III electrochemical detector and an ESA Coulochem data station (ThermoScientific, Waltham, MA, USA) was used to measure neurotransmitter levels including GABA, glutamate, dopamine (DA), 3,4-Dihydroxyphenylacetic acid (DOPAC), homovanillic acid (HVA), serotonin (5-HT), 5-hydroxyindoleacetic acid (5-HIAA), and norepinephrine (NE) in the brain tissues of SVZ, STR, HP, and OB.

Neurochemicals were separated on a Waters XBridge reverse-phase G18 column (150 × 3.0 mm, 3.5 μm particle size) (Water Corp, Milford, MA, USA). For monoamine separation, the mobile phase was: 80 mM NaH_2_PO_4_, 10% methanol, 2 mM octanesulfonic acid, 0.025 mM ethylenediaminetetraacetic acid and 0.2 mM trimethylamine, at pH 2.4. Monoamines were detected by analytical cell set at E1 = −150 mV and E2 = +350 mV. For quantification of GABA and glutamate, the mobile phase was 0.1 M Na_2_HPO_4_, 22% methanol and 4% acetonitrile, at pH 6.75. Quantification of amino acid neurotransmitters was accomplished through derivatization. The samples were mixed with a derivatization agent containing 0.M o-phthaladehyde (OPA), 0.05% 2-mercaptoethanol, 10% methanol in OPA diluent, prior to separation. Neurotransmitters in the sample were detected by analytical cell set at E1 = −150 mV and E2 = +550 mV. Levels of neurotransmitters tested here were calculated using area under the curve by comparison with standard curve. Levels were normalized to total protein amount and expressed as ng neurotransmitter/mg protein. DA and 5-HT turnover rates were also calculated.

### 2.9. Quantitative Real-Time RT-PCR

The transcription levels of mRNA encoding *Ctr1*, *Dmt1*, *Mt1a*, *Mt2a*, *Mt3*, *Gfap*, *Nestin*, *Dcx*, *NeuN*, *Th*, and *Gad67* were quantified using qPCR. The forward and reverse primers for all selected target genes and reference gene were designed using Primer Express 3.0 software. The sequences for all primers are listed in Table 1. All primers were obtained from Integrated DNA Technologies Ltd. (Coralville, IA, USA). Total RNA was isolated from control, Cu-deficient and Cu-overload rat CP, SVZ, and OB tissues by using TRIzol reagent following the manufacturer’s directions. An aliquot of RNA (1 μg) was reverse-transcribed into cDNA using the BioRad iScript cDNA synthesis kit. The iTaq Universal SYBR Green Supermix was used for qPCR analyses. The amplification was run in the CFX Connect Real-Time PCR Detection System with an initial 3 min denaturation at 95 °C, the amplification program was followed by 40 cycles of 30 s denaturation at 95 °C, 10 s at 60 °C and 30 s extension at 72 °C. A dissociation curve was used to verify that the majority of fluorescence detected could be attributed to the labeling of specific PCR products, and to verify the absence of primer dimers and sample contamination. Each qPCR reaction was run in triplicate.

The relative mRNA expression ratios between groups were calculated using the delta-delta cycle time formulation. After confirming that the reference gene was not changed, the cycle time values of interested genes were normalized with that of the reference gene in the same sample, and then the relative ratio between control and treatment groups was calculated and expressed as relative increases by setting the control as 100%. The amplification efficiencies of target genes and the internal reference were examined by determining the variations of the cycle time with a series of control template dilutions. Experimental conditions were optimized for annealing temperature, primer specificity, and amplification efficiency.

### 2.10. Statistical Analysis

All data are presented as mean ± SEM. Statistical analyses of the differences among control, CuD, and CuO groups were carried out by one-way ANOVA with a Tukey’s post hoc test. Since behavioral data were not normally distributed, this set of data was analyzed using the Mann–Whiney U-test (difference between two groups) or the Kruskal–Wallis nonparametric ANOVA (differences among three groups) followed by a Dunn’s test. All the statistical analyses were conducted using GraphPad Prism 6 software (GraphPad, San Diego, CA, USA). The differences between two means were considered significant at *p* ≤ 0.05.

## 3. Results

### 3.1. Systemic Cu Dyshomeostasis and Impaired Olfactory Function Following CuD or CuO Treatment

CuD and CuO treatments did not cause significant changes in rat body weight compared to controls (data presented in Figure S1). To demonstrate if treatment with CuD (AIN-93G CuD diet + 10 mg/kg TTM daily injection for 2 weeks) and CuO (normal AIN-93 diet + 3 mg Cu/kg daily injection for 2 weeks) (Figure 1A) caused systemic Cu dyshomeostasis, AAS was utilized to quantify Cu concentrations in the CSF, serum, and plasma. Cu levels in the CSF, serum, and plasma of the CuD rats were significantly lower than controls, while significantly higher in the CuO-treated animals as compared to controls (*p* < 0.05; Figure 1B). Further, our data revealed that blood Cu levels for all groups were more than one magnitude higher than their corresponding CSF Cu levels, indicating Cu homeostasis in the central milieu is tightly regulated by the blood-brain interfaces.

**Figure 2:**
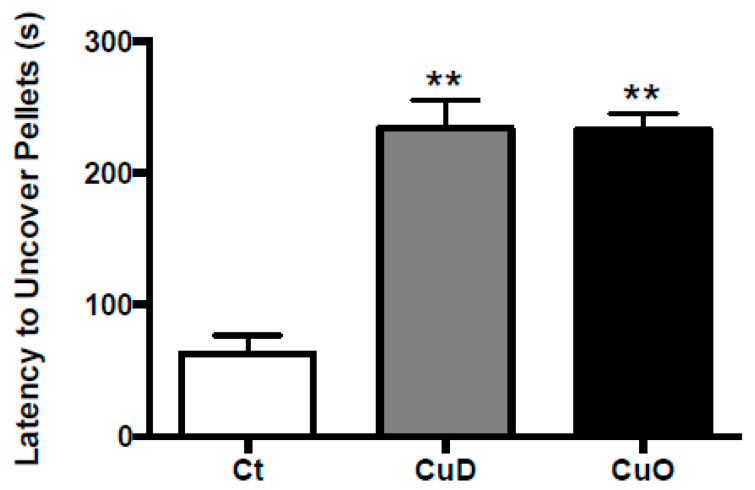
Buried pellet test to determine olfactory function. Rats were treated with CuD or CuO as described in Figure 1A. Tests were performed at the end of CuD or CuO treatment following three-day training and diet restriction. Data represents mean ± SEM, n = 14/group. **: *p* < 0.01, as compared to the control group, using Kruskal-Wallis nonparametric ANOVA analysis. Ct: control; CuD: Cu deficiency; CuO: Cu overload.

Upon confirmation of treatment-induced systemic Cu dyshomeostasis, a behavioral study was conducted to examine whether altered systemic Cu homeostasis affected olfactory function. Specifically, olfactory function was assessed by the buried pellet test among the three groups. The latency to uncover buried pellet was similar in both CuD and CuO rats, about 240 s; however, these values were significantly longer than that of control rats (about 60 seconds) (*p* < 0.01, Figure 2). In other words, CuD- or CuO- animals took approximately 4 times longer than controls to find the food pellet, suggesting that both Cu deficiency and overload conditions are capable of impairing olfactory sensory function.

### 3.2. Altered Brain Regional Cu Levels Following CuD or CuO Treatment

**Figure 3:**
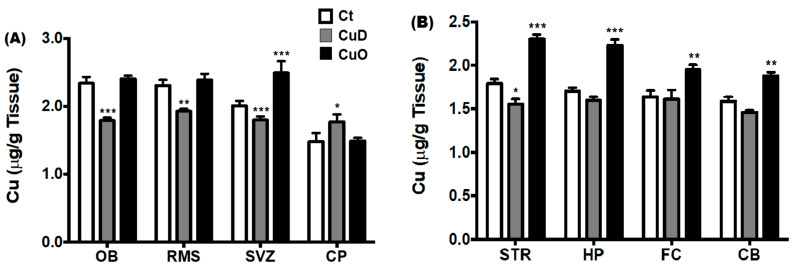
Brain regional Cu concentrations following CuD or CuO treatment. Brain tissues were dissected and analyzed for Cu by AAS. Data represents mean ± SEM, n = 6–7/group. *: *p* < 0.05, **: *p* < 0.01, ***: *p* < 0.001, as compared to the correspondent Ct group, using one-way ANOVA analysis. (**A**). Cu levels in the SVZ-RMS-OB axis and choroid plexus. (**B**). Cu levels in selected brain regions. Ct: control; CuD: Cu deficiency; CuO: Cu overload; CSF: cerebrospinal fluid; OB: olfactory bulb; RMS: rostral migratory stream; SVZ: subventricular zone; CP: choroid plexus; STR: striatum; HP: hippocampus; FC: frontal cortex; CB: cerebellum.

Previous studies from our laboratory have determined high Cu accumulation within the adult rat SVZ and OB [23,61], suggesting a crucial role of Cu in regulating adult neurogenesis, particularly in the SVZ-RMS-OB axis. As the first step to understand Cu-mediated mechanisms of olfactory dysfunction, we measured Cu levels in the SVZ-RMS-OB regions (i.e., OB, RMS, SVZ and choroid plexus), to compare to other brain regions (i.e., striatum (STR), hippocampus (HP), frontal cortex (FC), and cerebellum (CB)), in CuO, CuD, and control rats. Our data revealed that the 2-week CuD treatment markedly reduced Cu levels in the OB, RMS horizontal arm (olfactory tract), and SVZ (Figure 3A). The 2-week CuO treatment only increased the Cu levels in the SVZ, but had no effect on Cu levels in OB and RMS (*p* < 0.001, Figure 3A).

Interestingly, a significant increase of Cu was found in the choroid plexus of CuD-treated rats (*p* < 0.05), which was opposite to the general decreases observed in the OB, RMS, and SVZ (Figure 3A). As a barrier between the blood and CSF, it is possible that the blood-CSF barrier of the choroid plexus, in response to a decline in systemic Cu in CuD, may retain or slow down the loss of Cu from the CSF to maintain CSF Cu homeostasis.

Altered systemic Cu homeostasis could also affect Cu levels in other brain regions such as in the STR, HP, FC, and CB. Our data showed that the 2-week CuD treatment caused a significant reduction of Cu only in STR (*p* < 0.05 compared with controls, Figure 3B), but not in other tested brain regions. In contrast, the 2-week CuO treatment caused significant Cu increases in all four tested brain regions as compared to controls (*p* < 0.01, Figure 3B). It appeared as though regions associated with olfactory function and adult neurogenesis (i.e., OB, RMS, SVZ) tended to be more sensitive to the CuD treatment than other brain regions. Conversely, other brain regions tested (i.e., STR, HP, FC and CB) appeared to be more sensitive to the CuO treatment than the SVZ-RMS-OB regions.

### 3.3. Altered Expressions of Neuronal Markers Following Systemic CuD or CuO Treatment

**Figure 4:**
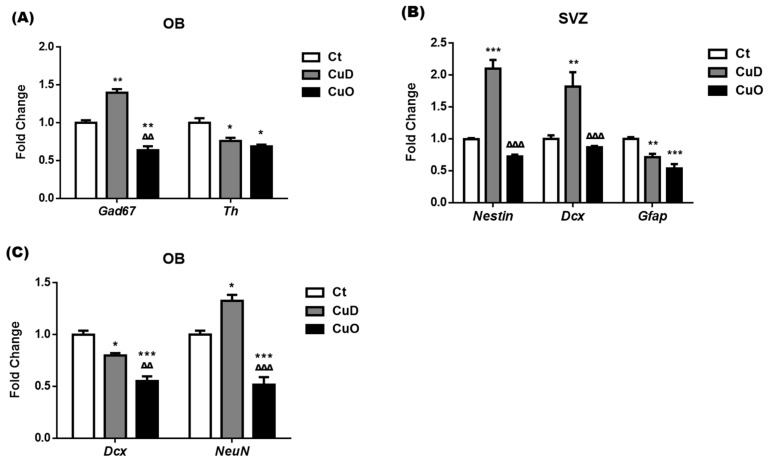
mRNA expression of neuronal markers following CuD or CuO treatment. The expression of neural markers was quantified by qPCR. (**A**). mRNA expression of Gad67 and Th in the OB. (**B**). mRNA expression of Nestin, Dcx, and Gfap in the SVZ. (**C**) mRNA expression of Dcx and NeuN in the OB. Data represents mean ± SEM, n = 6–7/group. *: *p* < 0.05, **: *p* < 0.01, ***: *p* < 0.001, as compared to the correspondent Ct group; ∆∆: *p* < 0.01, ∆∆∆: *p* < 0.001, as compared to the correspondent CuD group; using one-way ANOVA analysis. Ct: control; CuD: Cu deficiency; CuO: Cu overload; SVZ: subventricular zone; OB: olfactory bulb.

The adult OB circuits consist of diverse types of neurons residing in each layer; among them, two neuronal types of particular interest are GABAergic neurons expressing glutamic acid decarboxylase protein 67-kDa isoform (GAD67) and dopaminergic neurons expressing tyrosine hydroxylase (TH) [62]. Our qPCR data showed that the expression of *Gad67* mRNA in the OB was significantly upregulated by 40% and downregulated by 36% after the CuD and CuO treatment, respectively, when compared with controls (*p* < 0.01, Figure 4A). Treatments with either CuD or CuO, however, significantly reduced mRNA expression of *Th* in the OB, about 24% and 31% as compared to controls, respectively (*p* < 0.05, Figure 4A). Hence, the disrupted Cu homeostasis appeared to affect the neuron populations in the adult OB circuits, which could influence the secretion of neurotransmitters and alter normal olfactory function.

New neurons in the adult OB are originated from the SVZ, where adult neural stem cells (i.e., Type-B cells expressing cellular marker *Gfap*) proliferate to generate rapidly dividing transient amplifying cells (i.e., Type-C cells expressing marker *Nestin*), which give rise to neuroblast cells (i.e., Type-A cells expressing *Dcx*); these neuroblasts arrive the OB via the RMS and further differentiate into mature neurons (expressing marker *NeuN*) in OB [28,62,63,64,65]. To distinguish and identify the phenotypes of interested neural stem cells, neuroblasts, and neurons in the OB as influenced by disrupted adult neurogenesis in the SVZ-RMS-Ob axis, qPCR was used to quantify the expression of cellular mRNA markers of these neural stem and progenitor cells in the SVZ and OB.

Our data showed that the mRNA expression of *Nestin* and *Dcx* in the SVZ was significantly upregulated by 2.1- and 1.8-fold, respectively, following 2-week CuD treatment as compared to controls (*p* < 0.01, Figure 4B); however, the CuO treatment did not affect mRNA expression of *Nestin* and *Dcx* in the SVZ (Figure 4B). These alterations in *Nestin* and *Dcx* suggest that the CuD condition stimulates the proliferation of Type-C transient cells and Type-A neuroblasts, while these stem cells appear to tolerate CuO.

Interestingly, both CuD and CuO treatments significantly suppressed the mRNA expression of *Gfap* in the SVZ, a marker for Type-B progenitor cells, as compared to controls (*p* < 0.01, Figure 4B). The results indicate that a balanced Cu homeostasis is essential to maintain the normal proliferation behavior of the abundant GFAP(+) Type B stem cells in the SVZ.

In the OB tissue, both in vivo Cu deficient and overload treatments resulted in a marked inhibition in the mRNA expression of neuroblast marker *Dcx* by 20% and 45%, respectively (*p* < 0.05, Figure 4C). Noticeably, the neuroblasts that were upregulated in the SVZ (Figure 4A) did not seem to arrive or survive in the OB after CuD treatment (Figure 4C). In comparison to control rats, the *NeuN* mRNA expression level in the OB after CuD treatment was significantly increased by 33%, and yet a significant reduction of *NeuN* mRNA expression by 48% was observed in the OB of CuO-treated animals (*p* < 0.05, Figure 4C). These findings imply that an altered systemic Cu status may cause aberrant regulation of multiple processes contributing to disruption of proliferation, migration, and possibly differentiation of NeuN(+) neurons from the SVZ-originated DCX(+) neuroblasts in the OB.

### 3.4. Altered NSPC Proliferation in Adult SVZ Following Systemic CuD or CuO Treatment

**Figure 5:**
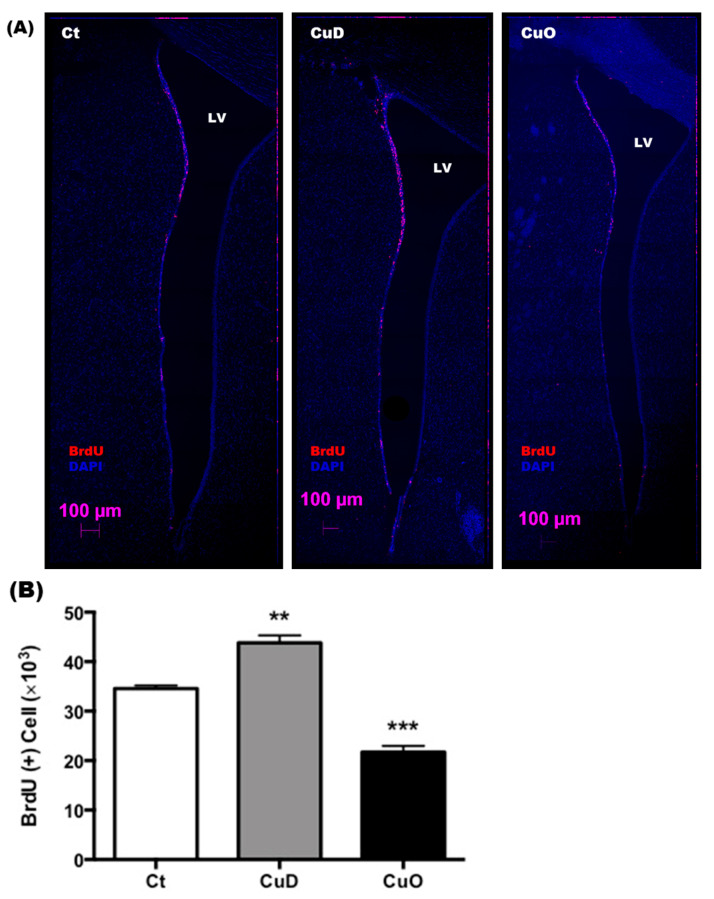
Confocal imaging study of proliferating NPSCs in the SVZ following CuD or CuO treatment. (**A**). Representative confocal images of the left coronal SVZ with BrdU (red signal)/DAPI (blue signal) staining. Scale bar = 250 µm. (**B**). BrdU(+) cell counts in the SVZ. Data represent mean ± SEM, n = 3–4/group. **: *p* < 0.01, ***: *p* < 0.001, as compared with the correspondent Ct group; using one-way ANOVA analysis.

To directly observe the impact of systemic Cu disorders on the proliferation of neural stem progenitor cells in the adult SVZ, CuD and CuO rats received a single ip. injection of the proliferation marker BrdU (100 mg/kg). Brain tissues were dissected 4 h later to trace newly generated proliferating cells (Figure 1A). Confocal images demonstrated the number of BrdU(+) proliferating cells alongside the SVZ were increased in CuD animals compared to controls; whereas BrdU(+) cells were reduced in the SVZ of CuO compared to controls (Figure 5A).

By counting BrdU(+) proliferating cells in SVZ, our results showed that CuD and CuO had the opposite effect by promoting and reducing the proliferation of NSPCs, respectively (*p* < 0.01, Figure 5B). Thus, this evidence supports the view that Cu acts on a yet-to-be-identified regulatory mechanism by controlling adult neurogenesis in the SVZ, ultimately affecting olfactory function.

### 3.5. Neurochemical Imbalance Following Systemic CuD or CuO Treatment

**Figure 6:**
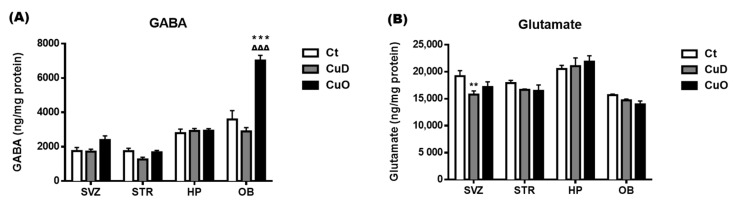
Changes of GABA and glutamate levels in selected brain regions following CuD or CuO treatment. Brain neuroactive amino acids were measured by HPLC. (**A**). Brain regional GABA levels. (**B**) Brain regional glutamate levels. Data represents mean ± SEM, n = 6–7/group. **: *p* < 0.01, ***: *p* < 0.001, as compared to the correspondent Ct group; ∆∆∆: *p* < 0.001, as compared with the correspondent CuD group; using one-way ANOVA analysis. Ct: control; CuD: Cu deficiency; CuO: Cu overload; SVZ: subventricular zone; STR: striatum; HP: hippocampus; OB: olfactory bulb.

Neuronal active chemicals such as GABA, glutamate, and catecholamines are vital in regulating olfactory function [66,67]. To investigate the influence of systemic Cu dyshomeostasis on the neurochemical balance of specific brain regions, a well-established HPLC method was used to determine levels of GABA, glutamate, and monoamines (i.e., DA, DOPAC, NE, 5-HT, 5-HIAA, and HVA) in the SVZ and OB in comparison to the striatum and hippocampus, following the 2-week CuD or CuO treatments. GABA levels of the CuD-treated animals were determined to be largely unchanged; the same results were observed in the CuO-treated animals, except for a marked increase, about two folds, in the OB, as compared to controls (*p* < 0.001, Figure 6A). Although no statistical significance was detected, there appeared to be an increased trend of GABA levels in the CuO-treated SVZ (Figure 6A).

The levels of glutamate in all tested brain regions under either CuD or CuO condition did not change significantly, except for a significantly lower glutamate level in the SVZ of CuD rats (*p* < 0.01, Figure 6B).

**Figure 7:**
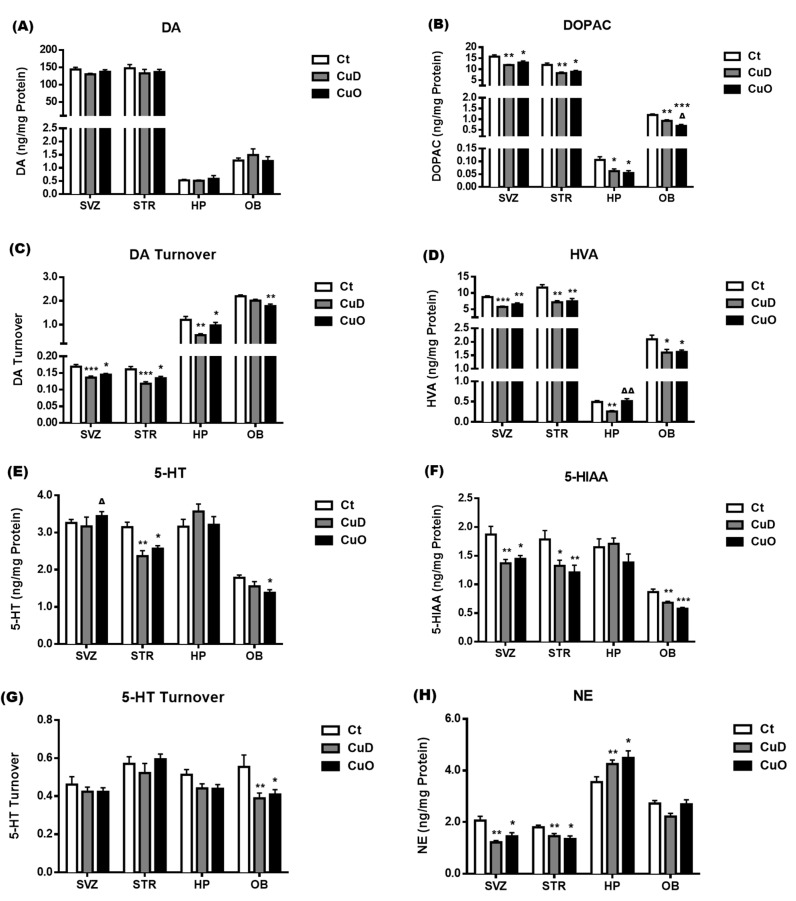
Changes of monoamine levels in selected brain regions following CuD or CuO treatment. Brain monoamine levels were assayed by HPLC. (**A**). Brain regional DA levels. (**B**). Brain regional DOPAC levels. (**C**). DA turnover in different brain regions. (**D**). Brain regional HVA levels. (**E**). Brain regional 5-HT levels. (**F**) Brain regional 5-HIAA levels. (**G**). 5-HT turnover rates in different brain regions. (**H**). Brain regional NE levels. Data represents mean ± SEM, n = 6–7/group. *: *p* < 0.05, **: *p* < 0.01, ***: *p* < 0.001, as compared with the correspondent Ct group; ∆: *p* < 0.05, ∆∆: *p* < 0.01, as compared with the correspondent CuD group; using one-way ANOVA analysis. Ct: control; CuD: Cu deficiency; CuO: Cu overload; SVZ: subventricular zone; STR: striatum; HP: hippocampus; OB: olfactory bulb; DA: dopamine; DOPAC: 3,4-Dihydroxyphenylacetic acid; HVA: homovanillic acid; 5-HT: 5-hydroxytryptamine or serotonin; 5-HIAA: 5-hydroxyindoleacetic acid; NE: norephinephrine.

Among the monoamines examined, neither CuD nor CuO treatment had any significant impact on the DA levels (Figure 7A), while both treatments significantly reduced DOPAC levels, as compared to controls (*p* < 0.05, Figure 7B). Interestingly, both DA and DPOAC contents were at similar levels in the SVZ and striatum, which were a magnitude higher than those in the hippocampus and OB (Figure 7A,B). The turnover rates of DA were significantly reduced in both of the CuD- and CuO- treated rats, as compared to controls, in all tested brain regions except for the OB of CuD-treated rats (*p* < 0.05, Figure 7C). In addition, the DA turnover rates in the hippocampus and OB were about one magnitude higher than those in the SVZ and striatum, which differed from the opposite magnitude pattern observed in DA and DOPAC (Figure 7A vs. Figure 7C). Similar to the pattern in DOPAC (Figure 7B), the levels of HVA, a major catecholamine metabolite, were significantly reduced in all tested brain regions following either CuD or CuO treatment, except for the CuO-treated hippocampus, as compared to controls (*p* < 0.05, Figure 7D). These findings indicate that systemic Cu dyshomeostasis disturbs DA pathways by interrupting DOPAC balance.

5-HT status was also quantified in the selected brain regions. Following CuD or CuO treatment, a significant increase in 5-HT was observed in the CuO-treated SVZ (*p* < 0.05, Figure 7E). However, both treatments resulted in a marked reduction in striatum 5-HT levels, while CuO treatment reduced OB 5-HT levels (*p* < 0.05, Figure 7E). The levels of 5-HIAA, a major metabolite of 5-HT, in the SVZ, OB and striatum were also significantly reduced, as compared to controls, following both CuD and CuO treatments (*p* < 0.05, Figure 7F). Noticeably, only in the OB were the turnover rates of 5-HT significantly slower than those of the control OB in both CuD and CuO treated animals (*p* < 0.05, Figure 7G). Taken together, these findings imply that the systemic Cu dyshomeostasis disrupts 5-HT balance and its metabolism in the SVZ-OB axis of the rat brain.

The systemic CuD and CuO treatments also significantly reduced NE levels in the SVZ and striatum but did not affect NE levels in the OB (*p* < 0.05, Figure 7H). Interestingly, NE levels in the hippocampus were much higher than in other tested brain regions, and treatment with either CuD or CuO significantly increased NE levels in the hippocampus as compared to controls (*p* < 0.05, Figure 7H). These NE alterations induced by systemic Cu disorders suggest that a stable Cu homeostasis may attribute to maintaining the balance of NE in the brain.

### 3.6. Altered Expression of Cu Transport-Associated mRNA Following Systemic CuD or CuO Treatment

**Figure 8:**
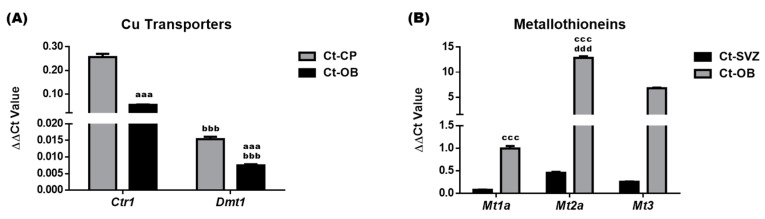
mRNA expression of Cu regulatory proteins in control rats. Expressions of mRNA encoding Cu regulatory proteins were quantified by qPCR. (**A**). ∆∆Ct values of Cu transporters Ctr1 and Dmt1 in the control CP and OB. (**B**). ∆∆Ct values of metallothioneins Mt1a, Mt2a, and Mt3 in the control SVZ and OB. Data represents mean ± SEM, n = 6–7/group. aaa: *p* < 0.001, as compared with the Ct-CP; bbb: *p* < 0.001, as compared with the correspondent Ctr1 ∆∆Ct values of the same tissue; ccc: *p* < 0.001, as compared with the Ct-SVZ; ddd: *p* < 0.001, as compared with the correspondent Mt1a ∆∆Ct values of the same tissue. SVZ: subventricular zone; OB: olfactory bulb.

Cu levels in the CSF and brain regions are regulated by Cu transporters and intracellular Cu binding proteins [39]. Altered Cu levels following CuD and CuO treatments observed in Figure 3 could result from the up- or down-regulation of these Cu regulatory proteins. Thus, we used qPCR to quantify Cu transporters (i.e., CTR1, DMT1) and Cu binding proteins (i.e., metallothionein isoforms MT1a, MT2a and MT3) in the SVZ, OB and choroid plexus, a tissue that is immediately adjacent to the SVZ and regulates Cu transport between the blood and CSF. After normalizing with the internal reference *Actb*, the delta-delta cycle time (∆∆Ct) values of *Ctr1*, *Dmt1*, *Mt1a*, *Mt2a*, and *Mt3* in these selected brain regions were presented in Figure 8. In control animals without treatment, the choroid plexus expressed *Ctr1* and *Dmt1* mRNA at a level more than 3–5 times higher than the expression level in the control OB; moreover, *Ctr1* mRNA levels were about 17-fold and 7-fold higher than *Dmt1* in the control plexus and OB tissues, respectively (*p* < 0.001, Figure 8A). The high mRNA abundance of the Cu transporting protein CTR1 in the choroid plexus suggests that CTR1 is essential in the transport and regulation of Cu ions between the blood and CSF, which may influence the Cu content of brain regions.

The mRNA expression level of metallothionein proteins (MT1a, MT2a and MT3) in the control OB were 12-, 28-, and 26-times higher than the expression level detected in the control SVZ, respectively (*p* < 0.001, Figure 8B). Such enhanced levels indicated that the OB could accumulate higher a level of Cu ions than the SVZ. Among those three metallothionein genes in both control SVZ and OB tissues, Mt2a had the highest mRNA expression, followed by Mt3 and Mt1a (Figure 8B), implying that MT2a and MT3 serve as the key Cu storage protein in both tissues.

**Figure 9:**
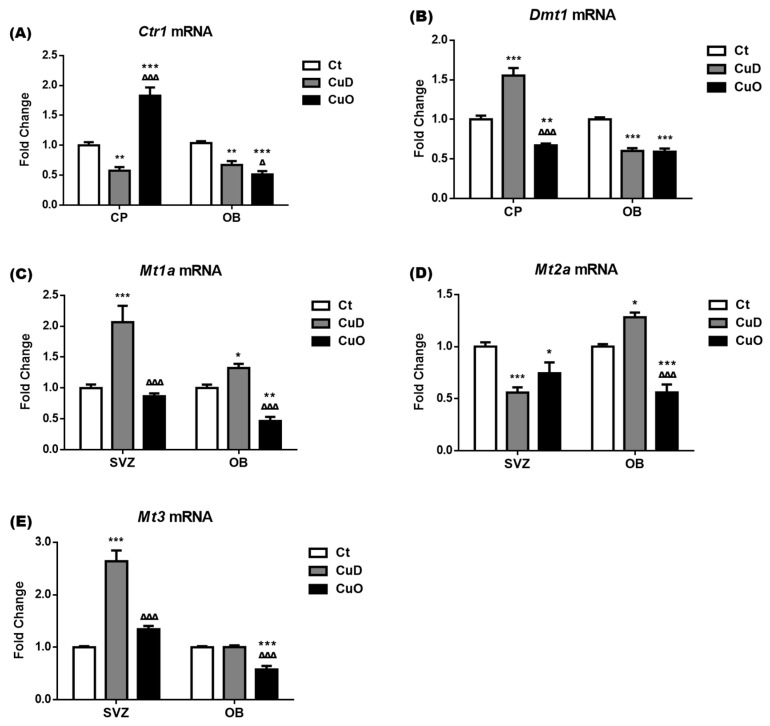
Expression of Cu regulatory mRNAs following CuD or CuO treatment. Expressions of mRNAs encoding Cu regulatory proteins were quantified by qPCR. (**A**). Ctr1 mRNA expressions in the choroid plexus (CP) and OB. (**B**). Dmt1 mRNA expressions in the CP and OB. (**C**). Mt1a mRNA expressions in the SVZ and OB. (**D**) Mt2a mRNA expressions in the SVZ and OB. (**E**) Mt3 mRNA expressions in the SVZ and OB. Data represents mean ± SEM, n = 6–7/group. *: *p* < 0.05, **: *p* < 0.01, ***: *p* < 0.001, as compared to the correspondent Ct group; ∆: *p* < 0.05, ∆∆∆: *p* < 0.001, as compared with the correspondent CuD group; using one-way ANOVA analysis. Ct: control; CuD: Cu deficiency; CuO: Cu overload; SVZ: subventricular zone; OB: olfactory bulb.

The 2-week CuD and CuO treatments significantly downregulated and upregulated *Ctr1* mRNA expressions by 42% and 83% in the choroid plexus, respectively, as compared to controls (*p* < 0.01, Figure 9A). In contrast to the transcriptional changes of *Ctr1*, the expression of *Dmt1* mRNA in the choroid plexus was markedly upregulated and downregulated after CuD- and CuO treatments, respectively (*p* < 0.01, Figure 9B). Specifically in the OB, both *Ctr1* and *Dmt1* mRNA expressions were downregulated under the CuD- or CuO- condition (*p* < 0.01, Figure 9A,B). These findings suggest that CTR1 and DMT1 appear to respond to CuD and CuO conditions in an entirely different manner in the blood-CSF barrier provided by the choroid plexus. Further, systemic CuD and CuO disrupt Cu transporters not only in Cu transporting tissue, i.e., the choroid plexus, but also in the OB which regulates olfactory function.

The 2-week CuD treatment significantly upregulated the mRNA expression of *Mt1a* and *Mt3* in the SVZ by 2.1 and 2.6 folds respectively, as compared to the control SVZ (*p* < 0.001, Figure 9C,E). However, *Mt2a* mRNA expression in the SVZ was significantly downregulated by both CuD and CuO treatments (*p* < 0.05, Figure 9D). In the OB, CuD treatment significantly induced the mRNA expression of *Mt1a* and *Mt2a* by 32% and 28%, respectively, without affecting the expression of *Mt3*, while the CuO treatment resulted in significant downregulation of all three genes by 53% in *Mt1a*, 44% in *Mt2a*, and 42% in *Mt3*, as compared to those of controls (*p* < 0.05, Figure 9C–E). The downregulation of *Ctr1*, *Dmt1*, *Mt1a*, *Mt2a*, and *Mt3* in the OB of CuO animals seems likely to indicate that this is a mechanism to regulate and maintain local Cu homeostasis in the OB under the systemic Cu overload condition, which is in agreement with AAS data where no significant Cu accumulation was observed in the OB of CuO rats (Figure 3A).

## 4. Discussion

The question of whether distorted Cu homeostasis affects olfactory function has long been unanswered. The current in vivo study provides first-hand evidence that systemic Cu dyshomeostasis, specifically Cu deficiency or Cu overload, likely impairs olfactory function by diminishing the animal’s natural sensation of smelling and food-finding. This impaired olfactory function under Cu dyshomeostasis is believed to be due, at least in part, to the disruption of Cu-mediated regulation of adult neurogenesis in the SVZ-RMS-OB axis. This conclusion is supported by several lines of observations. First, both CuD and CuO result in treatment-dependent reduction or elevation of Cu levels in local brain regions critical to olfactory function. Second, the locally altered Cu levels in the OB, RMS and SVZ apparently disrupt populations of GABA and DA neurons in OB, along with altering the expression of cellular markers for proliferation, migration, and differentiation of neural stem and progenitor cells in the SVZ-RMS-OB axis. Third, CuD and CuO treatments cause abnormal changes in neuroactive amino acids and monoamines in critical brain areas that regulate olfactory function. Finally, in vivo Cu dyshomeostasis clearly impairs mechanisms whereby Cu in the central milieus is regulated by transporters across brain barriers and storage proteins in local tissues. These observations support an essential role of Cu in adult neurogenesis and pertinent normal olfactory function.

Olfactory impairments, such as deficits in odor identification, detection, and discrimination, are found in patients with PD and AD [35,36,68,69]. Particularly with regards to PD, the olfactory dysfunction is one of the initial symptoms occurring years before motor symptoms and cognitive deficiency become evident. Thus, olfactory dysfunction has been suggested as a clinical indicator of the early stages of PD and also as an indicator of disease progression aiding in diagnosis [70]. The role of Cu in regulating olfactory function in healthy and disease conditions remains largely unknown. However, a few studies utilizing zebrafish models have provided the initial evidence to support Cu exposure and ensuing impaired olfaction. Specifically, they demonstrate Cu-mediated loss of behaviors critical to fish involving olfaction, such as predator avoidance, prey capture, mate selection, social behavior and migration [71,72,73].

Works by Baldwin et al., [71] report a Cu-dose-dependent inhibition of olfactory-mediated behaviors that are critical for the survival and migratory success of wild salmonids. By using the electro-olfactography to measure the level of olfactory dysfunction in zebrafish models, Dew et al. [74] reveal that exposure to a low, ecologically relevant Cu concentration causes a significant inhibition of olfactory activities. Julliard et al. (1996) also observe that olfactory deficits in zebrafish caused by Cu exposure can trigger aberrant olfactory neurogenesis, affecting both mature and immature neurons; the authors conclude that these responses are related to receptor-mediated cell death in the OB. Our observation of diminished olfactory function following systemic Cu dyshomeostasis in rats is in a good agreement with these previous reports from fish studies. Although little is known about the precise location of Cu in the OB, previous studies have shown that the mammalian OB has high concentrations of zinc (Zn) and Cu compared to other CNS regions [75,76]. Further, Zn is identified to exist primarily in olfactory sensory neuron terminals in the glomerular layer and neuron terminals in the granule cell layer [77,78,79]. With the close companionship that exists between Zn and Cu, Cu would likely concentrate within the olfactory sensory neurons as well. In addition, in vitro studies have observed that Cu influences the excitability of rat OB neurons by multiple mechanisms such as eliminating GABA-mediated spontaneous inhibitory postsynaptic potential, blocking the spontaneous glutamate-mediated excitatory synaptic activity, inhibiting sodium channels, and delayed rectifier-type potassium channels, etc. [80,81,82,83].

Several mechanisms may underscore Cu’s disruptive impact on olfactory function. The altered Cu level in critical brain regions relevant to olfaction (i.e., OB, SVZ, RMS) may cause alterations in adult neurogenesis in these areas affecting olfaction. This was suggested based on increases in proliferating markers in the SVZ of CuD and inhibition of these markers in the SVZ of CuO. It is possible that the loss of olfactory neurons may trigger adult neurogenesis in the SVZ-RMS-OB system by increasing the proliferation of olfactory NSPCs to replace the damaged sensory neurons in the OB. A zebrafish study demonstrates that Cu exposure can cause olfactory dysfunction and induce abnormal neurogenesis [4]. Moreover, a recent study by Ma et al. [84] demonstrates that transgenic zebrafish with diminished olfactory sensory neurons display an increased BrdU labeling after Cu exposure. Their further olfactory behavioral analyses reveal that the initial loss comes with a later restoration of olfactory neurons, which likely compensates for Cu-induced olfactory dysfunction. Our current study extended the research focus beyond the OB by examining responses throughout the entire SVZ-RMS-OB axis. Our data clearly establishes that the impact of systemic Cu disorder is not solely limited to the OB, but rather adversely affects processes of neurogenesis from its origin in the SVZ and along the SVZ-RMS-OB axis.

Noticeably, the Cu concentration in the SVZ is about 20–30 times higher than other brain regions, CSF, and plasma [19,23]. In our previous publications, we hypothesized that such a high Cu status in the SVZ may be necessary to maintain adult neurogenesis in this proliferative niche [19,24,61]. Although the amount of Cu necessary to regulate proliferation, differentiation, and migration of NSPCs in the SVZ-RMS-OB axis remains unknown, our current data appear to suggest that a disrupted Cu homeostasis impacts adult neurogenesis. Specifically, a reduced Cu status in the SVZ, as a result of CuD, may directly activate adult neurogenesis in the SVZ. This postulation was supported by the observation that Nestin(+) Type-C transit-amplifying cells and DCX(+) Type-A migratory neuroblasts were elevated following CuD treatment. In contrast, under high Cu conditions such as in the CuO animals, proliferation of GFAP(+) Type-C astrocytic stem cells and differentiation of NSPCs to NeuN(+) immature neurons were inhibited in the OB, pointing to an impaired adult neurogenesis. These observations prompt a new hypothesis that Cu in the SVZ functions as a biological “switch” that regulates adult neurogenesis. Specifically, adult neurogenesis is activated when Cu levels are low and inhibited when Cu levels are high. The molecular and cellular mechanisms by which Cu acts on its putative ligands thus warrant further investigation.

Our data determined an increased DCX expression in the SVZ and yet a reduced DCX expression in the OB of CuD rats. This could be due to unsuccessful arrival and/or reduced survival of SVZ-originated DCX(+) neuroblasts in the OB following CuD treatment. However, the possibility that some of the SVZ-originated neuroblasts may have differentiated into mature neurons at the time of experimentation cannot be ruled out. In fact, the latter hypothesis is partly supported by the observation of increased NeuN expression in the OB of the same CuD animals. Nonetheless, our observations support the disrupted proliferation, migration, and neuronal cell populations in the adult SVZ-RMS-OB system following disruption of Cu homeostasis.

Abnormal changes in neuroactive amino acids and monoamines in brain regions critical to olfaction may underlie Cu dyshomeostasis-associated olfactory dysfunction. Especially compelling in the CuO condition is the large increase in olfactory GABA and decreases in monoamine neurotransmitters/metabolites which could impact olfactory dysfunction. Reports in literature have suggested that Cu is an essential cofactor for at least twelve mammalian enzymes including dopamine beta-mono-oxygenase and actively participates in the synthesis of catecholamines, neuropeptide, and tyrosinase [47,85]. Reduced dopamine has been associated with dietary Cu deficiency in humans [86]. In addition, Cu ions have been found in synapses where they are released into the synaptic cleft, bind to neurotransmitters, and regulate activities of postsynaptic receptors, such as GABA receptors [6,87,88]. Thus, it seems likely the neurochemical imbalance observed in the SVZ, striatum, and hippocampus in the current study may be a direct result of altered Cu levels in these brain regions with ensuing disruptions in yet-to-be-defined Cu-sensitive pathways. These alterations may, in turn, underscore the motor and/or neurobehavioral dysfunction following systemic Cu dyshomeostasis.

Overall, Cu dyshomeostasis produces complex changes in neurotransmission that need to be further investigated. Given the effects on neurotransmitter metabolites and turnover observed in many brain regions, it becomes critical to examine whether Cu dyshomeostasis directly affects enzymes involved in neurotransmitter metabolism and also whether neurotransmitter release is modulated. Here, our surprising finding is that both Cu overload and deficiency seemed to produce similar alterations in monoamine neurotransmitters. Thus, it may be that a metal imbalance (in this case Cu) drives changes in neurotransmitter release/metabolism rather than their absolute levels. Specifically, increased or decreased levels of Cu do not produce differential effects but cause similar effects in regards to neurotransmitters. Data indicates an optimum threshold requirement that could be affecting two processes simultaneously: neurotransmitter release and metabolism.

Systemic Cu dyshomeostasis was determined to influence Cu levels in distinct local brain regions. Under normal physiological conditions, Cu is strictly regulated by a complex regulatory system involving membrane-bound Cu transporters, intracellular Cu chaperons, Cu-binding storage proteins, and cuproenzymes [39]. Cu in the blood circulation is transported into the brain and CSF via the blood-brain barrier and blood-CSF barrier in the choroid plexus, respectively [39,41,56]. As a barrier between the blood and CSF, the choroid plexus regulates the CSF Cu homeostasis. A significant increase in Cu was observed in the plexus tissue of CuD-treated rats, while Cu levels in the OB, RMS, and SVZ decreased. This observation possibly indicates that the choroid plexus may respond to systemic CuD by retaining or reducing the loss of Cu to maintain Cu homeostasis in the CSF.

In the brain, Cu is unevenly distributed [13,89,90,91,92,93], varies among species [94,95,96], and changes during development and in neurodegenerative conditions [97,98,99,100]. Markedly higher levels of Cu are found in the substantia nigra, locus coeruleus, and hippocampus [91,101,102,103]. The current study shows a higher expression of Cu transport proteins in the choroid plexus, which regulates Cu transport between the blood and CSF, than other tested brain regions and a comparatively lower expression of Cu storage proteins. These observations confirm similar distribution patterns of Cu regulatory proteins in previous reports [19,104,105]. An increase in a major Cu storage protein MT3 in SVZ and MT2 in OB after CuD treatment may explain the higher Cu levels in these tissues, while a decreased MT2 and MT3 in the SVZ and OB following CuO treatment may underscore lower Cu levels in the SVZ and OB.

The current study has several limitations. First, determination of neuronal markers such as Dcx (for Type A migratory neuroblasts), Nestin (for Type C transit-amplifying cells), GFAP (for Type B astrocytic stem cells), and NeuN (for mature neurons) is a valid approach to reflect the status of adult neurogenesis in the SVZ-RMS-OB axis. Yet, a more direct approach pertains to counting specific cell types double-labeled with BrdU. Second, the changes of a group of Cu transport and storage proteins were assayed by qPCR. In our future studies, it is desirable to conduct confocal experiments to verify alterations in the expression of these Cu regulatory proteins.

Finally, the phospholipids, mainly phosphatidylcholine and phosphatidylethanolamine, account for 81% (by weight) of the total lipid in rat olfactory mucosa, playing an indispensable role in maintaining the normal olfaction [106]. Recent data have suggested that chronic CuO in rats causes brain redox imbalance by generating free radicals via phospholipid peroxidation [107]. The evidence to suggest an interaction between Cu dyshomeostasis and phospholipid disruption in OB remains elusive; however, such a hypothesis along with changed endocannabinoid levels deserves further exploration.

In summary, our results from the animal models of Cu deficiency and Cu overload demonstrate systemic Cu dyshomeostasis can cause olfactory dysfunction. Further, mechanistic investigation suggests altered Cu status in local brain areas critical to olfaction may disrupt adult neurogenesis in the SVZ-RMS-OB axis, which supplies newly proliferated and differentiated neurons to the OB. These observations provide the first evidence to support a critical role of Cu in adult neurogenesis and in regulating olfactory function.

## Figures and Tables

**Table 1:** The forward and reverse primer sequences of target genes.

Category	Gene	Primer Sequence
Reference Gene	*Actb*	Forward: 5′-AGCCATGTACGTAGCCATCC-3′
		Reverse: 5′-CTCTCAGCTGTGGTGGTGAA-3′
Cu Transporter	*Ctr1*	Forward: 5′-TCGGCCTCACACTCCCACGA-3′
		Reverse: 5′-CGAAGCAGACCCTCTCGGGC-3′
	*Dmt1*	Forward: 5′-TCGCAGGCGGCATCTTGGTC-3′
		Reverse: 5′-TACCGAGCGCCCACAGTCCA-3′
Cu Binding Proteins	*Mt1a*	Forward: 5′-GCCTTCTTGTCGCTTACACC-3′
		Reverse: 5′-AGGAGCAGCAGCTCTTCTTG-3′
	*Mt2a*	Forward: 5′-ACAGATGGATCCTGCTCCTG-3′
		Reverse: 5′-GAGAACCGGTCAGGGTTGTA-3′
	*Mt3*	Forward: 5′-CCCTGCAGGATGTGAGAAGT-3′
		Reverse: 5′-TTTGCTGTGCATGGGATTTA-3′
Neuronal Markers	*Gfap*	Forward: 5′-TAGCATAAGTGGAGAGGGAA-3′
		Reverse: 5′-GGATTCAGAGCCAAGTGTAA-3′
	*Nestin*	Forward: 5′-ATGAGGGGCAAATCTGGGAA-3′
		Reverse: 5′-CCAGGTGGCCTTCTGTAGAA-3′
	*Dcx*	Forward: 5′-ACTGAATGCTTAGGGGCCTT-3′
		Reverse: 5′-CTGACTTGCCACTCTCCTGA-3′
	*NeuN*	Forward: 5′-TTCCCACCACTCTCTTGTCC-3′
		Reverse: 5′-GCAGCCGCATAGACTCTACC-3′
	*Th*	Forward: 5′-CAGGGCTGCTGTCTTCCTAC-3′
		Reverse: 5′-GGGCTGTCCAGTACGTCAAT-3′
	*Gad67*	Forward: 5′-CACAAACTCAGCGGCATAGA-3′
		Reverse: 5′-CTGGAAGAGGTAGCCTGCAC-3′

Note: The PCR primer efficiencies for listed primers were between 90% and 97%.

## Data Availability

The datasets generated during and/or analyzed during the current study are available from the corresponding author on reasonable request.

## Supplementary Materials

The following are available online at https://www.mdpi.com/article/10.3390/biom11091315/s1, Figure S1: Record of rat body weights during the course of Cu treatment.
