# Supplementary material for: Systemic Copper Disorders Influence the Olfactory Function in Adult Rats: Roles of Altered Adult Neurogenesis and Neurochemical Imbalance
Source: Biomolecules. 2021 Sep 6;11(9):1315. doi: 10.3390/biom11091315 (PMC8471899; doi:10.3390/biom11091315)
Supplement: Supplementary file 1 [file biomolecules-11-01315-s001.zip › biomolecules-1351175-supplementary.pdf]

Supplemental Materials

Systemic Copper Disorders Influence the Olfactory Function in Adult Rats: Roles of Altered Adult Neurogenesis and Neurochemical Imbalance

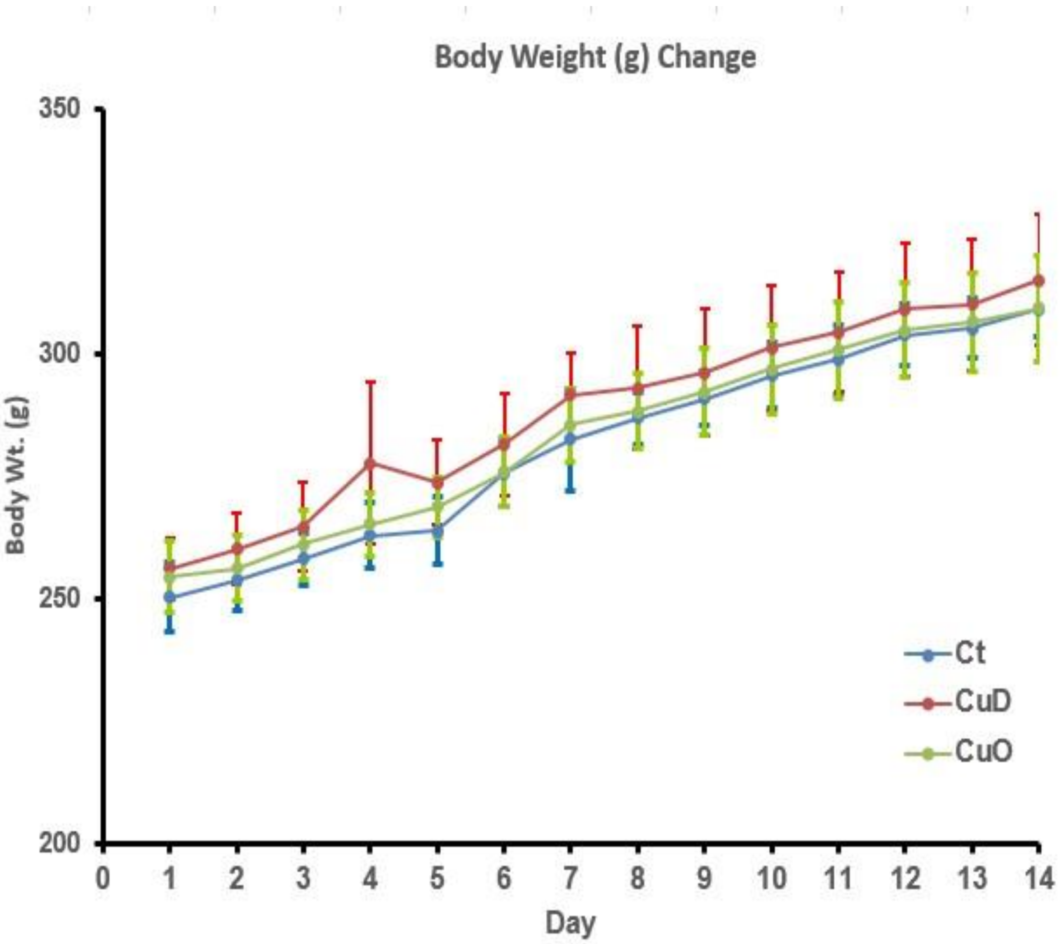

Figure S1. Record of rat body weights during the course of Cu treatment. Ct: control; CuD: Cu deficiency; CuO: Cu overload. Data represent mean ± SD, n = 14/group.
